# Manganese Mineralization of Pathogenic Viruses as a Universal Vaccine Platform

**DOI:** 10.1002/advs.202303615

**Published:** 2023-10-22

**Authors:** Pan‐Deng Shi, Yan‐Peng Xu, Zhu Zhu, Chao Zhou, Mei Wu, Yangzhige He, Hui Zhao, Liying Liu, Linqing Zhao, Xiao‐Feng Li, Cheng‐Feng Qin

**Affiliations:** ^1^ State Key Laboratory of Pathogen and Biosecurity Beijing Institute of Microbiology and Epidemiology Academy of Military Medical Sciences Beijing 100071 China; ^2^ Laboratory of Virology Beijing Key Laboratory of Etiology of Viral Diseases in Children Capital Institute of Pediatrics Beijing 100020 China; ^3^ School of Medicine Tsinghua University Beijing 100091 China; ^4^ Department of Medical Research Center State Key Laboratory of Complex Severe and Rare Diseases Peking Union Medical College Hospital Chinese Academy of Medical Science & Peking Union Medical College Beijing 100730 China

**Keywords:** innate immune activation, manganese mineralization, novel vaccine, resist infection, virulence attenuation

## Abstract

Biomimetic viral mineralization improves viral vaccine stability and immunogenicity using inorganic metals such as Ca, Al, or Fe. Mn is a metal found in high concentrations in mammalian tissues; however, under natural or laboratory conditions, Mn mineralization by medical viruses has yet to be established. Herein, a single IAV particle is successfully encapsulated with manganese phosphate (MnP) under specific conditions using the human influenza A virus (IAV). MnP‐mineralized IAVs (IAV@Mn) exhibited physiochemical and in vitro properties similar to Ca‐mineralized IAVs. In animal models, IAV@Mn shows limited replication in immune‐competent cells and a significant attenuation compared to naïve cells. Moreover, a single‐dose vaccination with IAV@Mn induced robust humoral and cellular immune responses and conferred significant protection against a wild‐type IAV challenge in mice. Thus, Mn mineralization in pathogenic viruses provides a rapid and universal strategy for generating an emergency vaccine in response to emerging viruses.

## Graphical abstract

Mn mineralization of pathogenic viruses accomplishes a rapid and universal strategy to generates an emergency vaccine in response to the emerging viruses. As the model, IAV@Mn showed limited replication and significant attenuation phenotype in vitro and vivo. A single‐dose vaccination of IAV@Mn induced robust humoral and cellular immune response and confer significant protection against wild type IAV challenge in mice.

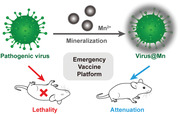

## 1 Introduction

Mineralization is a powerful tool for modifying viruses’ physicochemical and biological properties.^[^ ^1^ ^]^ Metals crystallize by settling on the surface of viral proteins.^[^ ^2^ ^]^ Amino acid residues provide multiple nucleation sites for depositing inorganic salt or metal nanoparticles.^[^ ^3^ ^]^ Because the virus's mineralized state is transitional, virus–inorganic hybrids are easily uncoated by cytomembranes, and viral infection occurs normally in the host.^[^ ^4^ ^]^ Mineral capsids prevent viral surface proteins from interacting with receptors, allowing viruses to avoid cellular barriers or immunological recognition.^[^ ^5^ ^]^ Furthermore, a compact shell structure can improve the virus's environmental stability while decreasing its inactivation efficiency.^[^ ^6^ ^]^ Currently, different metallic elements, including Ca, Al, and Fe, are used for virus mineralization for diverse purposes. For example, Ca‐mineralized influenza A virus (IAV) showed enhanced infectivity and transmissibility in mice,^[^ ^7^ ^]^ and Al‐gel nanocluster‐encapsulated enterovirus 71 (EV71) showed increased immunogenicity.^[^ ^8^ ^]^ Nevertheless, viral mineralization using specific inorganic minerals has multiple applications in biomedicine.

Mn is one of the most abundant metals in mammalian tissues and regulates Mn‐dependent enzymes, including oxidoreductases, isomerases, and hydrolases, in various physiological processes.^[^ ^9^ ^]^ Mn increases the sensitivity of the cGAS‐STING pathway to double‐stranded DNA (dsDNA) and is required for host defense against DNA viruses. After viral infection, Mn^2+^ is released from mitochondria and Golgi, accumulates in the cytoplasm, and binds to cGAS to promote STING activation. More importantly, Mn^2+^ is a potent cGAS activator that induces the production of type I interferons (IFN‐I) and cytokines in the absence of infection.^[^ ^10^ ^]^ Colloidal Mn salt (MnJ) was formulated as an adjuvant to stimulate humoral and cellular immune responses, inducing antibody production and CD4^+^/CD8^+^ T cell proliferation and activation by either intramuscular or intranasal immunization.^[^ ^11^ ^]^ This unique feature has broadened the potential applications of Mn in vaccine development.

An incident beam is used to observe the precipitation of Mn‐bearing minerals on bacterial surfaces in nature. The surface charges and exopolymer types of bacteria influence Mn nucleation site density and accessibility.^[^ ^12^ ^]^ However, whether Mn can mineralize viruses is unknown. Herein, we used Mn to broaden the concept of virus mineralization. Characterization of the resulting Mn‐encapsulated virus hybrids (Virus@Mn) revealed a physiochemically similar but biologically distinct feature from mineralized viruses with other inorganic materials.

IAVs are a large family of segmented para‐stranded RNA viruses that cause widespread morbidity and mortality due to human respiratory diseases. Although traditional inactivated vaccines against influenza A and B viruses are commercially available, their protective efficacy is greatly impaired by variations in the hemagglutinin (HA) and neuraminidase (NA) protein levels.^[^ ^13^ ^]^ Most importantly, the frequent reassortment of different subtypes of IAV generates novel IAV strains with pandemic potential,^[^ ^14^ ^]^ highlighting the importance of developing a rapid vaccine platform for these emerging viruses.

## 2 Results and Discussion

In this study, we first established an experimental system to realize Mn mineralization using IAV as a model. The traditional inorganic mineralization procedure was modified. The formation efficiency of mineralized products was determined by different concentrations of Mn^2+^ and PO_4_^3–^ solutions. Mn‐encapsulated IAVs (IAV@Mn) in the precipitate were tested using Mn^2+^ concentrations ranging from 0.01 to 100 mm. At 1 mm, excess Mn^2+^ did not improve mineralization efficiency (Figure S1a, Supporting Information). Simultaneously, high PO4^3−^ concentrations reduced IAV@Mn hybrids in the mineralization (Figure S1b, Supporting Information). As the pH decreased, IAV@Mn hybrids struggled to maintain their mineralized form, and the IAV gradually uncoated (Figure S1c, Supporting Information). The viral release was 100% at pH 5 (Figure S1d, Supporting Information). The optimal reaction conditions in the solution were 1 mm Mn^2+^ and 1 mm PO4^3−^ to complete mineralization of ≈95% IAVs.

**Figure 1:**
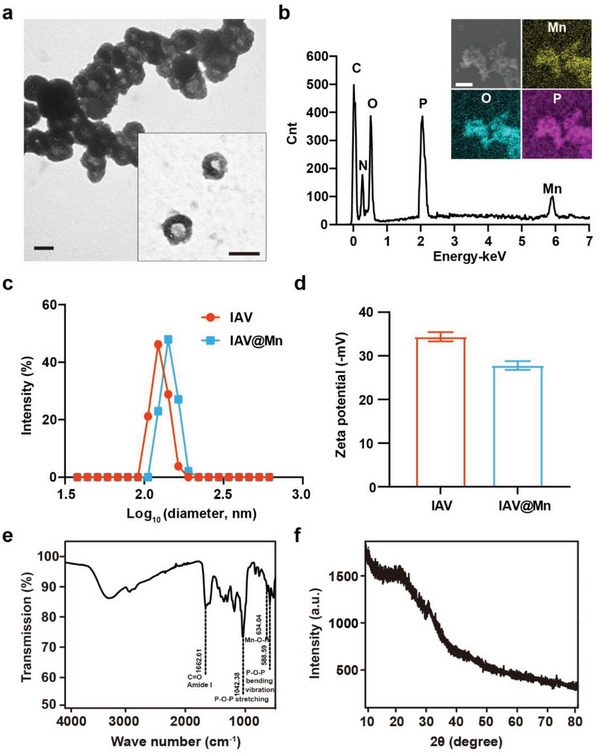
Physiochemical characterization of MnP‐encapsulated IAV. a) TEM image of IAV@Mn. Samples were negatively stained with phosphotungstic acid. Scale bar = 100 nm. b) EDS analysis and SEM image of IAV@Mn and in situ elemental mapping of Mn, P, and O on the nanoparticle surface. Scale bar = 1 µm. c) Mean diameter of IAV and IAV@Mn measured via DLS. d) Zeta potential of IAV and IAV@Mn measured via NTA. e) FTIR analysis of IAV@Mn; Mn─O─H bond was detected at 634.04 cm^−1^. f) XRD analysis of IAV@Mn.

The physicochemical and morphological properties of the prepared IAV@Mn hybrids were characterized and compared with those of naïve IAV. Under a transmission electronic microscope (TEM), the stained (phosphotungstic acid) IAV@Mn hybrids showed a darker outline and some aggregation (**Figure** **1**a), similar to the Ca‐mineralized IAV (Figure S2, Supporting Information). Scanning electron microscopy (SEM) and energy dispersive X‐ray spectroscopy (EDS) confirmed the presence of Mn, O, and P on the capsids of the nanoparticles through in situ elemental mapping. The surface elemental compositions revealed that the hybrids comprised Mn salts and organic matter (Figure 1b). Dynamic light scattering analysis indicated that the mineralized IAV's hydrodynamic diameter increased from 124 to 142 nm (Figure 1c). Surface charge analysis indicated that the zeta potential changed from −34.36 ± 1.04 to −27.79 ± 1 mV (Figure 1d), and Ca mineralization of IAV caused a similar change to −16.63 ± 1.39 mV. Fourier transform infrared spectroscopy (FT‐IR) detected a strong transmission peak at 1042.38 cm^−1^, which was the phosphate radical's asymmetric stretching vibration; the phosphate radical's in‐plane bending vibration was 588.99 cm^−1^, and Mn─O─H was detected at 634.04 cm^−1^ (Figure 1e), indicating the formation of a manganese phosphate (MnP) mineral phase on the IAV@Mn hybrid surface. X‐ray photoelectron spectroscopy (XPS) of precipitated IAV@Mn revealed that Mn^2+^ remained the dominant element (Figure S3, Supporting Information). X‐ray diffraction (XRD) analyses revealed that the exterior of MnP was almost amorphous (Figure 1f), similar to IAV@Ca.^[^ ^7^ ^]^ These findings confirmed that Mn can mineralize single viral particles under specific conditions.

To verify the versatility of the Mn mineralization strategy, enterovirus type 71 (EV71) and respiratory syncytial virus (RSV) were mineralized by Mn under similar conditions (Figure S4a, Supporting Information). Mn mineralizes both EV71 and RSV at different mineralization efficiencies. The zeta potential of EV71@Mn and RSV@Mn differed noticeably from the native ones (Figure S4b, Supporting Information). XRD analyses of EV71@Mn and RSV@Mn also revealed an amorphous shell (Figure S4c, Supporting Information).

**Figure 2:**
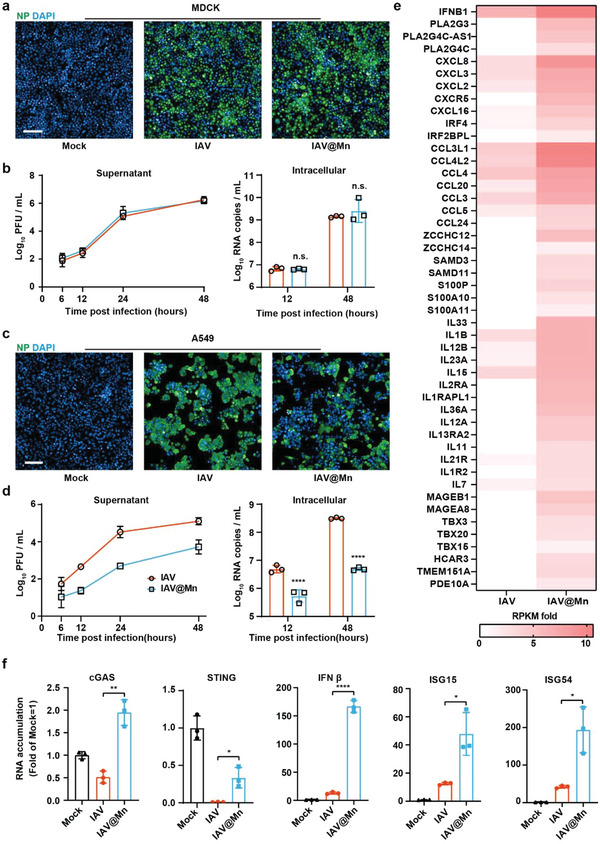
Attenuation of IAV@Mn in vitro. a) Indirect fluorescence assay of IAV and IAV@Mn in A549 cells (MOI = 0.01) at 48 h.p.i. Cell nuclei were stained by DAPI (blue), and the IAV NP was stained by IAV NP‐specific antibody (green). Scale bar = 100 µm. b) Growth curve of IAV and IAV@Mn in MDCK cells (MOI = 0.01). Virus in supernatant was detected using plaque assay, and the intracellular RNA accumulation was detected by qPCR. c) Indirect fluorescence assay of IAV and IAV@Mn in A549 cells (MOI = 0.01) at 48 h.p.i. d) Growth curve of IAV and IAV@Mn in A549 cells (MOI = 0.1). e) Heat map of RNA‐seq analysis. THP‐1 cells were infected with IAV or IAV@Mn at 0.1 MOI. The heat map was generated by calculating treated RPKM/control RPKM. f) mRNA transcription level analysis of cGAS and STING expression was detected at 6 h.p.i; IFN‐β, ISG15, and ISG54 expression were detected at 24 h.p.i. Cell experiment data are presented as mean ± SD, n = 3, and *P*‐values were calculated using one‐way or two‐way ANOVA with Bonferroni correction, *P<0.05, **P<0.01, ****P<0.0001.

Because Ca or Al mineralization enhances viral entry and replication in cell cultures, we further determined the effect of Mn mineralization on viral infectivity. Similar to the IAV@Ca hybrids, IAV@Mn could infect MDCK cells, resulting in typical cytopathic effects and plaque on the third day post‐infection (d.p.i.) (Figure S5, Supporting Information).^[^ ^15^ ^]^ An indirect fluorescence assay (IFA) confirmed that Mn materialization did not change the viral infection rate in MDCK cells (**Figure** **2**a). The dynamics of viral proliferation were measured using the infectious particles in the supernatant and intracellular viral RNA copies. Both IAV@Mn and IAV exhibited consistent growth patterns (Figure 2b). However, compared with naïve IAV, IAV@Mn showed significantly restricted replication in human A549 cells. The IFA results further demonstrated that the viral infection rates of IAV@Mn and IAV were different (Figure 2c). Viral RNA accumulation was distinguishable in the IAV@Mn‐infected cells (Figure 2d). IAV@Mn almost lost its infectivity, and no infectious virus was detected in the supernatant of human THP‐1 cells (Figure S6, Supporting Information), unlike the effect of Ca mineralization on IAV.^[^ ^7^ ^]^

To elucidate the underlying mechanism restricting IAV@Mn replication, we performed RNA‐seq analysis of the treated THP‐1 cells. The heat map of the transcriptome exhibited a cluster of IFN‐stimulated genes (ISGs), of which IAV@Mn infection upregulated 1.5‐fold relative to IAV infection (Figure 2e). The hybrids caused a more significant upregulation of innate immunity than naïve viral infection. The RNA transcription levels of cGAS‐STING pathway genes were validated using qPCR. Compared to IAV, IAV@Mn infection significantly promoted the activation of related genes (Figure 2d), indicating that the MnP capsid of the IAV@Mn hybrids activated the cGAS pathway by inducing the expression of downstream IFN‐related genes to inhibit viral proliferation.^[^ ^16^ ^]^

**Figure 3:**
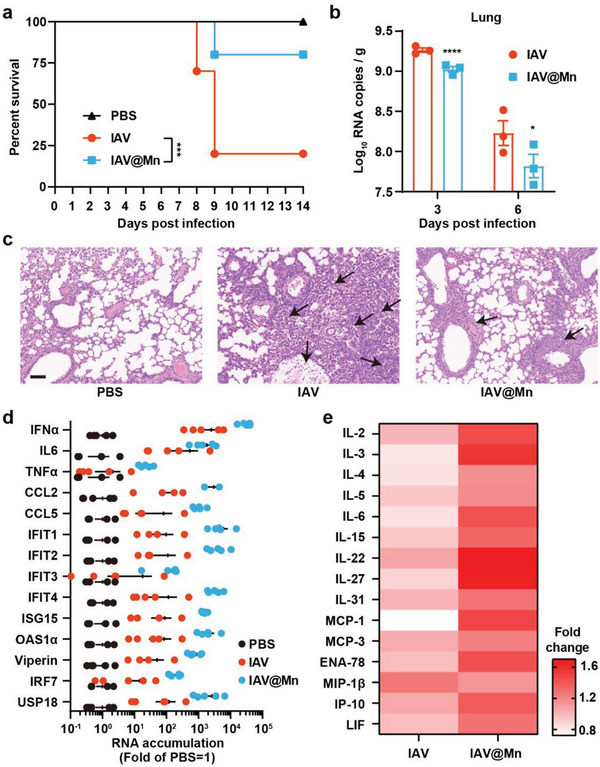
Attenuation of IAV@Mn in vivo. PBS was set as the control, and 5–6‐week‐old female BALB/c mice were intranasally infected with 2 × 10^4^ PFU IAV or IAV@Mn. a) Percent survival of mice (*n* = 10). Data are presented as mean ± SD, *n* = 10, and *P*‐values were calculated using a logrank test, ***P<0.001. b) Replication of IAV and IAV@Mn in the lungs of mice. Tissues were collected at 3 and 6 d.p.i. Data are presented as mean ± SD, *n* = 3, and *P*‐values are calculated using two‐way ANOVA with Bonferroni correction, *P<0.05, ****P<0.0001. c) H&E staining of lung pathology (20×). Arrows indicate pathological tissue. Scale bar = 50 µm. d) qPCR analysis of cGAS‐STING pathway in the lung at 24 h.p.i. RNA accumulation was calculated using GAPDH as a reference gene and folded by PBS as 1. Data are presented as mean ± SD, *n* = 4. e) Serum cytokine levels at 24 h.p.i. The results were detected using Luminex 200 (*n* = 3).

Next, we investigated the effect of Mn mineralization on viral pathogenicity in animals. In vivo, 5–6‐week‐old female BALB/c mice were intranasally infected with 2 × 10^4^ plaque‐forming units (PFU) of either IAV or IAV@Mn and monitored daily for 14 d for survival statistics and weight loss measurements. Mice with a body weight loss of >20% were recorded as dead for ethical reasons. The maximum weight loss in the IAV@Mn‐infected groups was 10% lower than that in the IAV‐infected group (Figure S7, Supporting Information). Within 14 d, 20% of the IAV@Mn‐infected mice were dead, considerably less than 80% of the IAV‐infected mice (**Figure** **3**a). Viral RNA in the lungs was detected by qPCR. Compared with the IAV‐infected group, viral replication was inhibited by Mn mineralization on days 3 and 6 (Figure 3b). After IAV infection, the lung tissue showed alveolar atrophy and a widened alveolar septum (black arrow) (Figure S8, Supporting Information), and numerous lymphocyte and granulocyte infiltrations emerged (black arrow). Necrotic cell debris appeared in the bronchial lumen along with blood vessel congestion and dilation (Figure 3c). The lymphocytes and granulocytes increased significantly, and connective tissue hyperplasia was the main pathological feature in IAV@Mn‐infected mice, indicating that IAV@Mn infection induced milder symptoms.

Furthermore, RNA accumulation of IFN pathway genes in the lungs was detected 24 h.p.i. Compared with the phosphate‐buffered saline (PBS)‐ and IAV‐treated groups, IAV@Mn significantly upregulated IFN‐related genes (Figure 3d), consistent with in vitro experiments. Simultaneously, the Luminex assay showed that IAV@Mn activated higher levels of inflammatory cytokines in the serum than the IAV infection (Figure 3e; Figure S9, Supporting Information). These results demonstrate that Mn mineralization significantly activates innate immune responses in mice.

**Figure 4:**
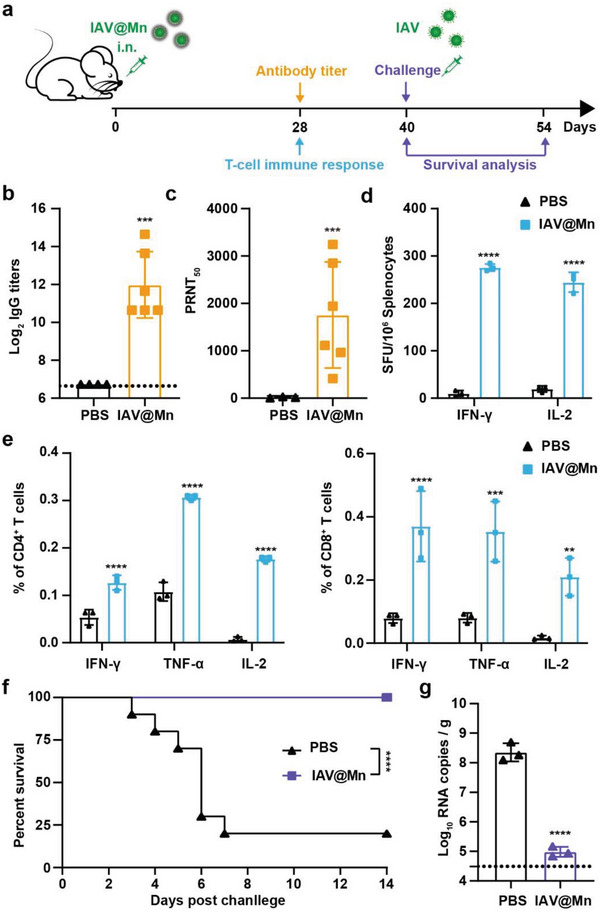
Immunogenicity and immune correlates of protection of IAV@Mn in mice. Three‐week‐old female BALB/c mice were intranasally infected with 10^4^ PFU IAV@Mn. PBS was used as the placebo. a) Schematic diagram of IAV@Mn immunization, sample collection, and immunological assays. b) IAV HA‐specific IgG antibody titers measured by ELISA. Data are presented as mean ± SD, *n* = 6, and *P*‐values were calculated using unpaired *t*‐tests, ***P<0.001. c) Neutralizing antibody titers measured by PRNT. Data are presented as mean ± SD, *n* = 6, and *P*‐values were calculated using unpaired *t*‐tests, ***P<0.001. d) Production of IFN‐γ and IL‐2 in splenocytes measured by ELISPOT. Data are presented as mean ± SD, *n* = 3, and *P*‐values were calculated using two‐way ANOVA with Bonferroni correction, ****P<0.0001. e) Production of IFN‐γ, TNF‐α, and IL‐2 in splenocytes measured by flow cytometry. Data are presented as mean ± SD, *n* = 3, and *P*‐values were calculated using two‐way ANOVA with Bonferroni correction, **P<0.01, ***P<0.001, ****P<0.0001. f) Percent survival of mice after 10^5^ PFU IAV challenge. Data are presented as mean ± SD, *n* = 10, and *P*‐values were calculated using a logrank test, ****P<0.0001. g) Replication of IAV in the lungs of mice at 6 d post‐challenge. Data are presented as mean ± SD, *n* = 3, and *P*‐values were calculated using unpaired *t*‐tests, ****P<0.0001.

Because of the unexpected attenuation of IAV@Mn in mice, we explored the possibility of developing IAV@Mn as a candidate vaccine. Briefly, BALB/c mice were intranasally immunized with IAV@Mn, and humoral and T‐cell immune responses were measured on day 28 (**Figure** **4**a). A single‐dose vaccination with IAV@Mn induced high levels of IAV‐specific immunoglobulin G (IgG) antibodies (Figure 4b). Neutralizing antibodies with a 50% plaque reduction neutralization titer (PRNT_50_) approached 1/1754 after immunization (Figure 4c). Enzyme‐linked immunosorbent spot assay (ELISPOT) and flow cytometry were used to detect the cytokines secreted by spleen cells in different groups of mice. ELISPOT showed that secretion of interferon γ (IFN‐γ) and interleukin‐2 (IL‐2) in splenocytes was significantly higher than in the groups that received PBS vaccination (Figure 4d). Flow cytometry revealed that IAV‐specific CD4^+^ and CD8^+^ effector T cells in splenocytes were effectively activated by IAV@Mn, which was stimulated with peptide pools covering the IAV HA protein (Figure 4e). Both parts demonstrated that IAV@Mn hybrids could be used as vaccine agents that elicit efficient humoral immunity and Th1‐biased T‐cell immunity in vivo via intranasal administration.

A lethal dose of IAV (10^5^ PFU) was used to evaluate the protective efficacy of this novel vaccine in vivo. Compared to the PBS group, IAV@Mn‐vaccinated mice showed improved survival rates from 20% to 100% (Figure 4f). Almost no virus was detected in the lung tissue after IAV reinfection (Figure 4g). IAV@Mn hybrids produced effective immune protection via intranasal administration, dependent on the activation of both Mn‐relative innate immunity and adaptive immunity induced by the limited viral replication.

## 3 Conclusion

In conclusion, we achieved a novel proof‐of‐concept for the instantaneous transformation of pathogenic viruses into vaccine candidates via rapid, economic, and feasible Mn mineralization. MnP hybrid formation expanded the new modality of viral mineralization, contrasting from that of traditional CaP. Chemical characterization showed that MnP induced a looser and softer mineralization layer than CaP did.^[^ ^7^ ^]^ Interestingly, IAV@Mn did not show improved thermostability compared to intact IAV (Figure S10, Supporting Information), in contrast to IAV@Ca.^[^ ^7^ ^]^ The underlying mechanisms accounting for this difference require further investigation.

In contrast to live‐attenuated vaccine technology, genetic modifications of viruses or long‐term passages have been omitted in Mn mineralization. In our trial system, the RNA viruses produced MnPs with differences in mineralization efficiency. To ensure viral infectious ability, MnP endowed attenuation characteristics to the hybrids owing to its special activation effect on innate immune signaling molecules. For respiratory viruses, early mucosal immune activation and formation of the innate immune barrier are effective in preventing viral infections.^[^ ^17^ ^]^ Therefore, local immune stimulation of the respiratory tract effectively attenuates virulence and provides an effective guarantee of the early protection efficacy of the vaccine. Meanwhile, the hybrids effectively induced robust antigen‐specific adaptive responses and immune memory, which lays a potential foundation for their value as vaccines.

## 4 Experimental Section

### Materials

*Mice*: BALB/c mice used in this study were purchased from the Animal Laboratory Animal Center, People's Liberation Army Academy of Military Medical Sciences, Beijing, China. The mice were pathogen‐free and maintained under a 12‐h light/dark cycle on a standard chow diet. All animal experiments were performed per the guidelines of the Chinese Regulations of Laboratory Animals (Ministry of Science and Technology of China) and were approved by the Institutional Animal Care and Use Committee at the Beijing Institute of Microbiology and Epidemiology (IACUC‐IME‐2021‐009).

*Cells and Viruses*: Human THP‐1 cells were a gift from Dr. Zhengfan Jiang of the School of Life Sciences, Peking University. The other cells were purchased from ATCC and stored in our laboratory. Human THP‐1 cells were cultured in RPMI‐1640 supplemented with 10% fetal bovine serum (FBS) at 37 °C in an incubator with 5% CO_2_. MDCK, A549, rhabdomyosarcoma (RD), and Hep2 cells were cultured in Dulbecco's Modified Eagle Medium (DMEM) containing 10% FBS at 37 °C in an incubator with 5% CO_2_.

IAV strain A/WSN/1933(H1N1) was grown in the allantoic cavities of 9‐day‐old embryonated hen's eggs for 48 h at 33 °C. The viruses were collected from the allantoic fluid and frozen at −70 °C until use. EV71 strain HN08/08 was prepared in RD cells when typical cytopathic effects developed 36–48 h post‐infection (h.p.i.) and frozen at −70 °C until use. RSV strain A2 was prepared in Hep2 cells when typical cytopathic effects developed 96–120 h.p.i. and frozen at −70 °C until use. Virus titers were determined using a plaque assay. All experiments using live viruses were performed in a biosafety level‐2 containment laboratory.

### Virus Mineralization and Characterization

*Mn Mineralization on Viruses*: Viral solutions (10^5^ PFU mL^−1^) in Opti‐MEM supplemented with 1 mm manganese chloride (MnCl_2_) were vortexed at 400 rpm at room temperature (RT) for 10 min. Subsequently, 1 mm sodium dihydrogen phosphate (Na_2_HPO_4_) was added to the solution dropwise with vortex oscillation at 400 rpm at RT for 10 min. After reaction completion, the mixture was centrifuged at 10 000 ×*g* for 10 min at 4 °C. The supernatant was discarded, and the sediment was suspended in fresh Opti‐MEM to prepare the final stock of Virus@Mn. Mineralization efficacy was calculated, viral RNA was detected using qPCR, and the CT value was converted into RNA copies.

(1) $$Mineralization\;efficacy\;=\frac{mineralized\;virus\;in\;the\;sediment}{virus\;before\;mineralization}\;\times 100\%$$

*Plaque‐Forming Assays*: Plaque‐forming assays of IAV, EV71, and Virus@Mn were performed using MDCK or RD cells. Briefly, cells were seeded in a 12‐well plate for 24 h and infected with 400 µL of tenfold viral dilutions. The IAV supernatant was replaced with DMEM containing complete FBS, 1% low‐melting point agarose (Promega, Madison, WI, USA), 1 mg mL^−1^ bovine serum albumin (BSA) (Sigma‐Aldrich, St. Louis, MO, USA), and 1 µg mL^−1^ TPCK (Sigma‐Aldrich). The EV71 supernatant was replaced with DMEM containing 2% FBS. Viral plaques were detected at 3–4 d.p.i. Cells were then fixed with 4% formaldehyde and stained with 1% crystal violet solution. Plaque numbers were recorded after rinsing the plates with deionized water, and the final titers were calculated accordingly.

*One‐Step Quantitative Real‐Time PCR (qPCR)*: RNA of samples was extracted using RNA Purelink RNA mini kit (Invitrogen, Waltham, MA, USA), and one‐step qPCR was used to quantify the amount of viral RNA using One Step PrimeScript RT‐PCR Kit (Takara Bio, San Jose, CA, USA) with IAV‐specific primers (forward, 5′‐GACCRATCCTGTCACCTCTGAC‐3′; reverse, 5′‐GGGCATTYTGGACAAAKCGTCTACG‐3′) and probe (5′‐FAM‐TGCAGTCCTCGCTCACTGGGCACG‐BHQ1‐3′), EV71‐specific primers (forward, 5′‐GGCCATTTATGTGGGTAACTTTAGA‐3′; reverse, 5′‐CGGGCAATCGTGTCACAAC‐3′) and probe (5′‐FAM‐AAGACAGCTCTCGCGACTTGCTCGTG‐BHQ1‐3′), and RSV‐specific primers (forward, 5′‐AGCAAATCAATGTCACTAGCACC‐3′; reverse, 5′‐TCATCAGTCTTTGTGGTGTGGTA‐3′) and probe (5′‐FAM‐AGCCAACCCAACCATGGACACAACCCA‐BHQ1‐3′). Absolute quantification of RNA was performed using a standard curve generated by serially diluting an RNA solution of the determined titer in RNase‐free water.

*Indirect Fluorescence Assay (IFA)*: Cells at 90%–100% confluences were infected with the virus at the desired multiplicity of infection (MOI). The infected cells were fixed at different time points post‐infection with precooled acetone at −20 °C for 30 min, washed with PBS, and then incubated with an IAV nucleoprotein (NP)‐specific antibody (GeneTex, Irvine, CA, USA) for 1 h. The cells were washed with PBS and incubated with goat anti‐mouse Alexa Fluor 488 conjugated secondary antibody (GeneTex). After washing the infected cells with PBS, DAPI (Sigma‐Aldrich) was added, and the cells were incubated at RT for 3 min for nuclear staining. Fluorescence was detected after washing cells with PBS. Images were acquired using an Opera automated microscope (PerkinElmer, Waltham, MA, USA).

*Histopathology Assay*: For histopathological analysis, mouse lung tissues were fixed in 4% neutral‐buffered formaldehyde for 48 h, embedded in paraffin, sectioned, and stained with hematoxylin and eosin (H&E). Images were captured using an Olympus microscope and analyzed using CaseViewer.

### Chemical Characterization

*TEM Characterizations*: The viral solutions were added onto carbon‐coated copper TEM grids (400 mesh; Agar Scientific) by dip‐coating, and the samples were dried at RT before observation. The samples were stained with phosphotungstic acid. TEM observations were performed using an HT‐7700 microscope (Hitachi, Tokyo, Japan).

*EDS*: The elemental mapping of IAV@Mn and the EDS spectra were investigated using an EDS attached to an SEM (TESCAN MIRA LMS, Czech Republic).

*DLS*: Size was measured using DLS on Zetasizer Nano‐ZS90 (Malvern Panalytical).

*Zeta Potential*: The zeta potential was determined at pH 7 using Nanoparticle Tracking Analysis (NTA) with ZetaVIEW S/N 20–568 (Particle Metrix). Data were analyzed using ZetaView software (version 8.05.14 SP7).

*FTIR*: The FTIR spectra of IAV@Mn were obtained using an FTIR spectrometer (Nicolet iS50; Thermo Fisher Scientific, Waltham, MA, USA).

*XPS*: XPS was performed using an XPS instrument (K‐Alpha; Thermo Fisher Scientific), and the data were fitted using the Avantage Data System (Thermo Fisher Scientific).

*XRD*: The crystalline structures of the samples were analyzed using XRD (SmartLab SE; Rigaku, Tokyo, Japan).

### Immunological Evaluation

*Enzyme‐Linked Immunosorbent Assay (ELISA)*: Serum IgG antibodies against IAV were detected using indirect ELISA in 96‐well flat‐bottomed plates (CoStar). The plates were coated with IAV HA (Sino Biological) in 0.1 m carbonate/bicarbonate buffer (pH 9.6) and incubated at 4 °C overnight. After blocking with 5% BSA in PBS and washing twice with PBST, the plates were incubated with double gradient diluted mice sera starting from 1:100 in duplicate wells for 1 h at 37 °C. The plates were washed thrice with PBST and incubated with diluted peroxidase‐conjugated goat anti‐mouse IgG (ZSGB‐Bio) in PBS at 37 °C for 1 h. After washing three times with PBST, 3,3′,5,5′‐tetramethylbenzidine (TMB) substrate was added. The absorbance of the plates was determined at 450 nm and corrected for the background using PBS as a control.

*Plaque Reduction Neutralization Test (PRNT)*: MDCK cells were seeded in 12‐well plates and incubated for approximately 16 h until 90%–100% confluent. Serum was prepared in DMEM by twofold serial dilutions starting from 1:100. The diluted sera were then mixed with virus in a 1:1 ratio to generate a mixture and incubated at 37 °C for 1 h. Virus/serum mixtures were added to the wells of 12‐well plates. The plates were then incubated at 37 °C for 1 h. The mixtures were removed, and the cells were overlaid with 1% low‐melting point agarose in DMEM. After further incubation at 37 °C for 2 d, the cells were fixed with 4% formaldehyde and stained with 1% crystal violet. Plaque numbers were recorded after rinsing the plates with deionized water. The 50% neutralization titer (PRNT_50_) was calculated using the Spearman–Karber method.

*Enzyme‐Linked Immunospot Assay (ELISPOT)*: Cellular immune responses in the vaccinated mice were assessed using IFN‐γ and IL‐2, precoated ELISpot kits (MabTech), according to the manufacturer's protocol. Briefly, the plates were blocked with RPMI 1640 containing 10% FBS and incubated for 30 min. Immunized mouse splenocytes were then plated at 300 000 cells/well, with a peptide pool for IAV HA protein (2 mg mL^−1^ of each peptide), concanavalin A (ConA, Sigma‐Aldrich) as a positive control, or RPMI 1640 medium as a negative control. After incubation at 37 °C and 5% CO_2_ for 36 h, plates were washed with PBST, and biotinylated anti‐mouse IFN‐γ and IL‐2,  antibodies were added to each well, followed by incubation for 2 h at RT. After adding a 3‐amino‐9‐ethylcarbazole (AEC) substrate solution, the air‐dried plates were read using an automated ELISPOT reader (AID). The number of spot‐forming cells (SFC) per 1000 000 cells was then calculated.

*Flow Cytometry Analyses*: T cell proliferation in immunized mice was evaluated using a FACSCalibur flow cytometer (BD Biosciences). Briefly, 1000 000 mouse splenocytes were stimulated with an IAV HA peptide pool (2 mg mL^−1^ of each peptide) for 2 h at 37 °C with 5% CO_2_. Brefeldin A (1 mg mL^−1^, MCE) was then added to splenocytes and incubated for 4 h. Following two washes with PBS, splenocytes were permeabilized and stained with fluorescently conjugated antibodies against CD3 (PE/Cyanine7) (BioLegend), CD4 (FITC) (BioLegend), CD8 (APC/FITC) (BioLegend), CD44 (PE) (BioLegend), or CD62L (APC) (BD Biosciences). Dead cells were stained using a Zombie UV3 Fixable Viability Kit (BioLegend). Data were analyzed with FlowJo software.

### Quantification and Statistical Analysis

All data were analyzed with GraphPad Prism 8.4 software. No statistical methods were used to predetermine the sample size unless indicated otherwise. The investigators were not blinded to the allocation during the experiments and outcome assessment unless indicated otherwise (qPCR). Unless specified, data are presented as mean ± SD in all experiments. Analysis of variance (ANOVA) or unpaired *t*‐test was used to determine the statistical significance among different groups (^*^ *P*<0.05; ^**^ *P*<0.01; ^***^ *P*<0.001; ^****^ *P*<0.0001; n.s., not significant).

## Conflict of Interest

The authors declare no conflict of interest.

## Supporting information

Supporting Information

## Data Availability

The data that support the findings of this study are available in the supplementary material of this article.
