# Supplementary material for: Manganese Mineralization of Pathogenic Viruses as a Universal Vaccine Platform
Source: Adv Sci (Weinh). 2023 Oct 22;10(33):2303615. doi: 10.1002/advs.202303615 (PMC10667830; doi:10.1002/advs.202303615)
Supplement: Supplementary file 1 — Supporting Information [file ADVS-10-2303615-s001.pdf]

ADVANCED SCIENCE
Open Access

Supporting Information

for *Adv. Sci.*, DOI 10.1002/advs.202303615

Manganese Mineralization of Pathogenic Viruses as a Universal Vaccine Platform

Pan-Deng Shi, Yan-Peng Xu, Zhu Zhu, Chao Zhou, Mei Wu, Yangzhige He, Hui Zhao, Liying Liu, Linqing Zhao, Xiao-Feng Li and Cheng-Feng Qin**

Supporting Information

Manganese mineralization of pathogenic viruses as universal vaccine platform

Supporting Information Figures

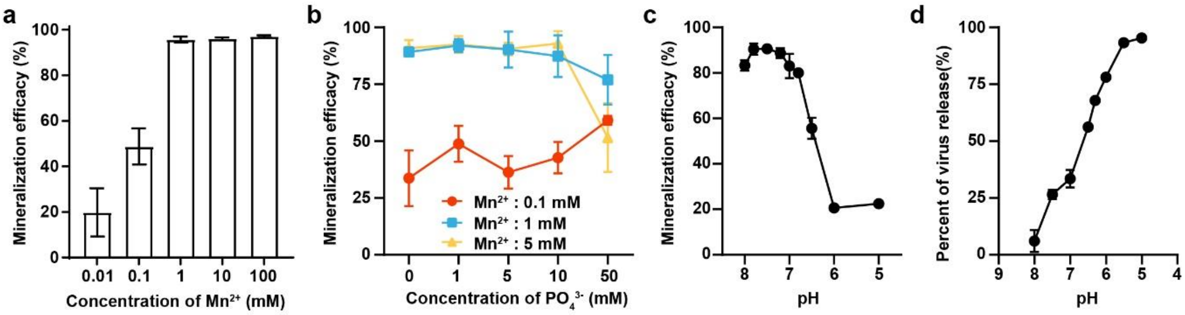

Figure S1. MnP mineralization efficiency under different conditions. Mn^{2+} (a), PO_4^{3-} (b) and pH (c) affected the formation of MnP hybrids. The mineralization efficacy was calculated as the number of virus particles before and after mineralization. d)The IAV@Mn in sedimentation were added to the solution with different pH, and centrifuged at 10000 g again to collect and measure the viruses that retained the mineralized shell.

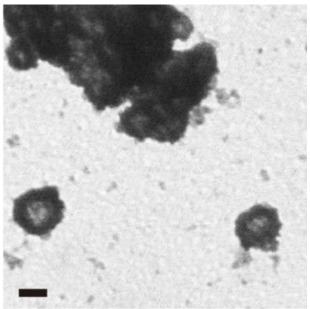

Figure S2. TEM image of IAV@Ca. Sample was negatively stained with phosphotungstic acid. Scale bar, 100 nm.

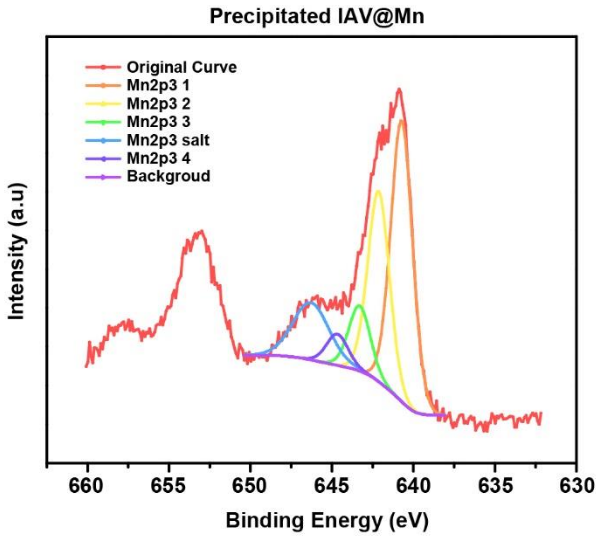

Figure S3. Valence state of Mn in precipitated IAV@Mn was detected by X-ray photoelectron spectroscopy (XPS). The samples were calculated by original curve (red) and the other fitted curve.

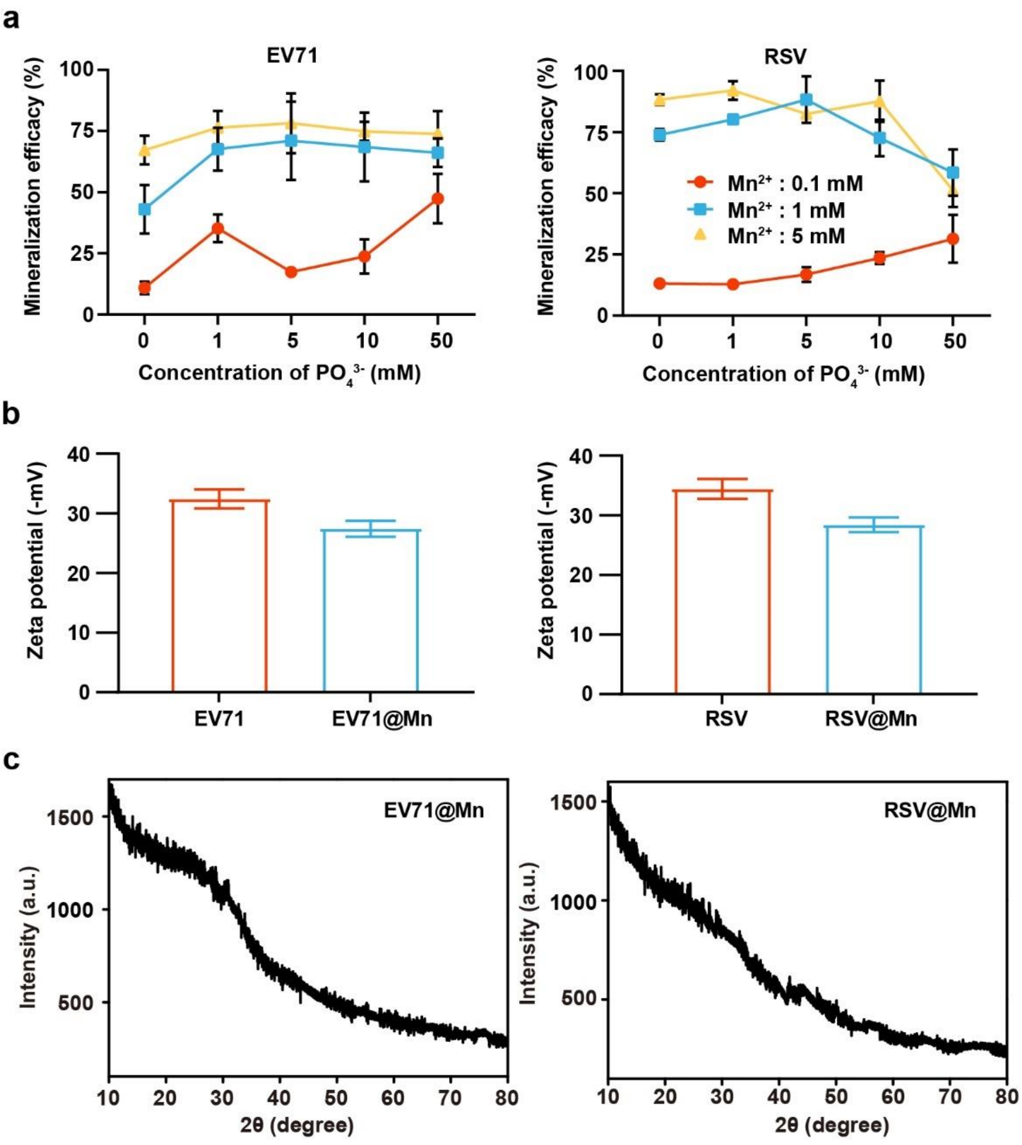

Figure S4. MnP mineralization of EV71 and RSV. a) concentration of Mn^{2+} and PO_4^{3-} affected the formation of MnP hybrids. b) Zeta potential of EV71@Mn and RSV@Mn measured via NTA. c) XRD analysis of EV71@Mn and RSV@Mn.

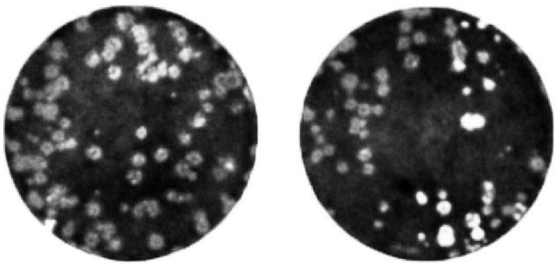

Figure S5. IAV@Mn was biodegradable to restore viral bioactivity. Plaque morphologies of IAV and IAV@Mn in MDCK cells.

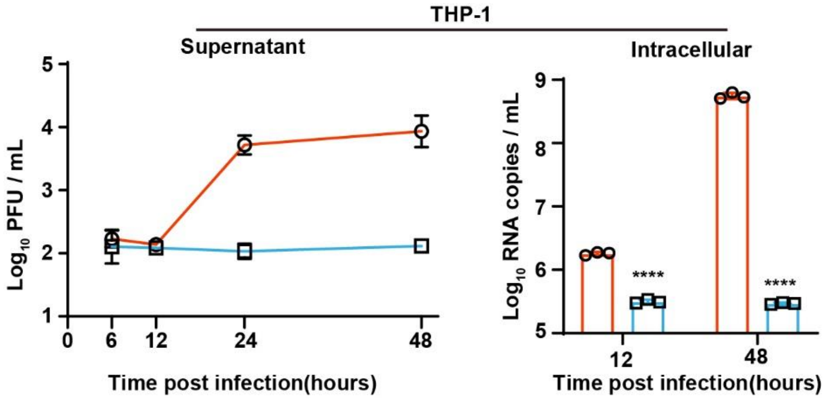

Figure S6. Growth curve of IAV and IAV@Mn in THP-1 cells (MOI=0.1). Virus in supernatant was detected by plaque assay and the intracellular RNA accumulation was detected by qRT-PCR. Cell experiment data are presented as mean ± SD, n=3, and *P*-values were calculated using one-way or two-way ANOVA with Bonferroni correction, ****P<0.0001.

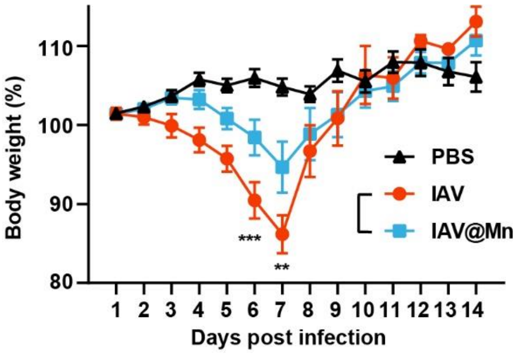

Figure S7. Body weight change of mice. 5-6 weeks female BALB/c mice were intranasally infected with 2×10^4 PFU IAV or IAV@Mn. Data are presented as mean ± SD, n=10, and *P*-values are calculated using two-way ANOVA with Bonferroni correction, $^{**}P<0.01$, $^{***}P<0.001$.

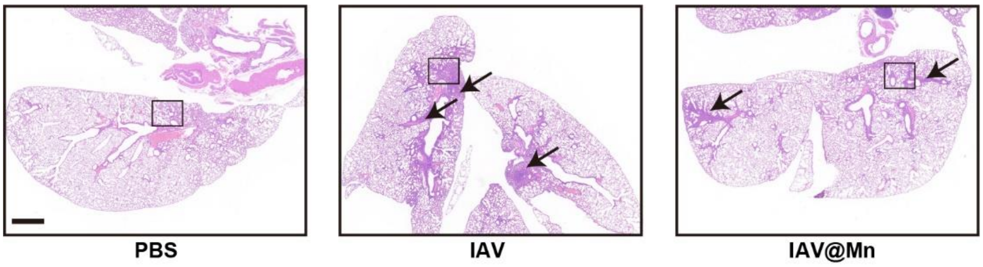

Figure S8. H&E staining of lung pathology (2×). The box indicated the part at 20×magnification. Arrows indicate pathological tissue. Scale bar, 1 mm.

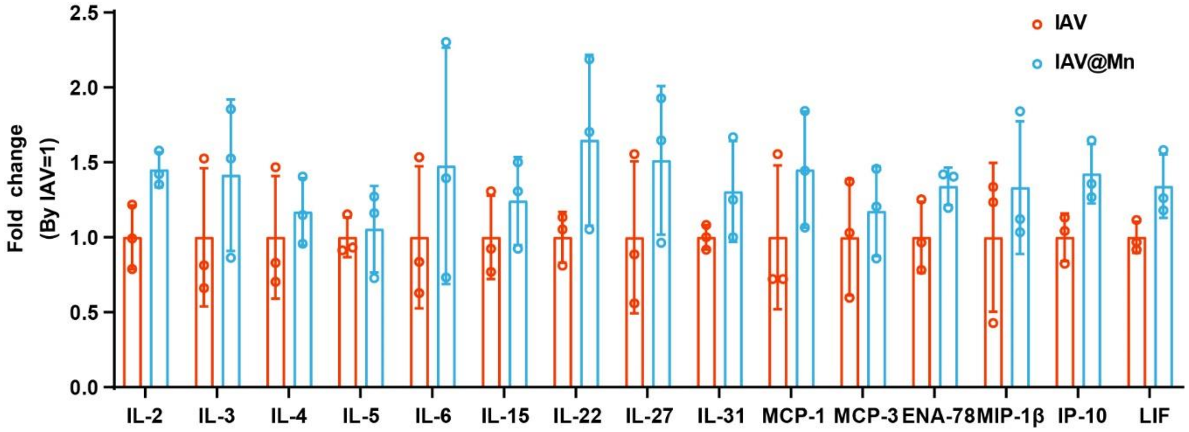

Figure S9. Levels of the cytokines in serum at 24 h.p.i. The data were detected by Luminex® 200TM.

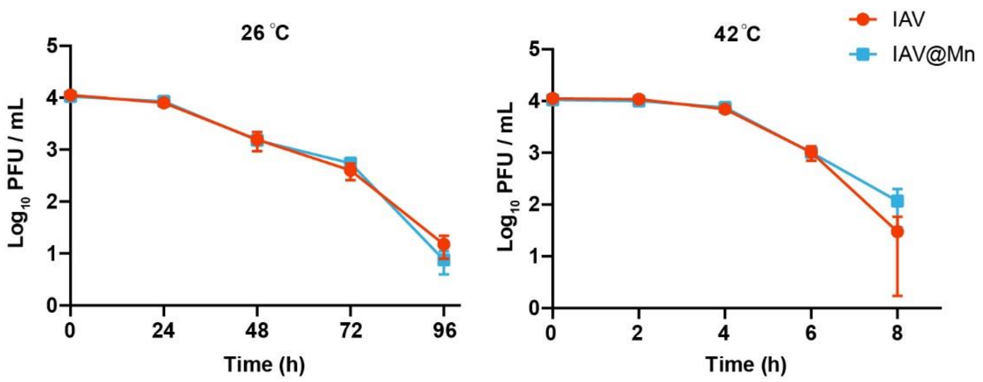

Figure S10. The thermostability of IAV and IAV@Mn at 26 ℃ and 42 ℃.

Table S1. Primers for qRT-PCR

Primers	Forward (5'- 3')	Reverse (5'- 3')
cGAS	CACGAAGCCAAGACCTCCG	GTCGCACTTCAGTCTGAGCA
STING	CCAGAGCACACTCTCCGGTA	CGCATTTGGGAGGGAGTAGTA
IFN β	ATGACCAACAAGTGTCTCCTCC	GGAATCCAAGCAAGTTGTAGCTC
ISG15	GAGAGGCAGCGAACTCATCT	CTTCAGCTCTGACACCGACA
ISG54	AAGCACCTCAAAGGGCAAAAC	TCGGCCCATGTGATAGTAGAC
IFNα	GAAGGACAGGAAGGATTTTGGA	TGAGCCTTCTGGATCTGTTGGT
IL6	TCCATCCAGTTGCCTTCTTG	GGTCTGTTGGGAGTGGTATC
TNFα	GGGTGATCGGTCCCCAAAGG	CTCCACTTGGTGGTTTGCTACGA
CCL2	CACTCACCTGCTGCTACTCA	GACCTTAGGGCAGATGCAGT
CCL5	CAAGTGCTCCAATCTTGCAGTC	TTCTCTGGGTTGGCACACAC
IFIT1	CTCTGAAAGTGGAGCCAGAAAAC	AAATCTTGGCGATAGGCTACGA
IFIT2	CTGAAGCTTGACGCGGTACA	ACTTGGGTCTTTCTTTAAGGCTTCT
IFIT3	TTCCCAGCAGCACAGAAAC	AAATTCCAGGTGAAATGGCA
IFIT4	ATTACAAAAGAAGACATGACAGAC	AGGCAAAACCAAAGACTCCA
ISG15	CTAGAGCTAGAGCCTGCAG	AGTTAGTCACGGACACCAG
OAS1α	TGAGCGCCCCCCATCT	CATGACCCAGGACATCAAAGG
Virepin	ATAGTGAGCAATGGCAGCCT	AACCTGCTCATCGAAGCTGT
IRF7	CAATTCAGGGGATCCAGTTG	AGCATTGCTGAGGCTCACTT
USP18	GAGAGGACCATGAAGAGGA	TAAACCAACCAGACCATGAG
GAPDH	AACTTTGGCATTGTGGAAGG	ACACATTGGGGGTAGGAACA
